# An Open Letter from "Ierne": To the Hon. Sir Arthur Stanley, G.B.E., M.P., Chairman of the College of Nursing

**Published:** 1918-05-18

**Authors:** 


### WHY WORKING NURSES SHOULD ARTICULATE.

### AN OPEN LETTER FROM "IERNE."

### To the Hon. Sir Arthur Stanley, G.B.E., M.P., Chairman of the College of Nursing.

Sir,—May I be permitted to address to you an open letter of friendly and appreciative criticism? The usual underlying motive of open letters in the press to public men is to win applause from the gallery for saying unpleasant things. I am inspired by no such desire. You are a champion of a great cause, and what I desire to say may not, I am afraid, be regarded as orthodox in the most select circles. The views that I strongly hold I wish to hold forth for your consideration and for the consideration of all whom they may concern, and without any attempt to dogmatise. Your endeavours to achieve betterment for British nurses are obviously honourable, honest, and patriotic, and therefore I regard it as a duty for anybody who can do so to help to speed the movement to success. This is my apology. I entertain, no doubt, that the future chronicler of the evolution of British nursing will unhesitatingly state that since Florence Nightingale, by her devotion and self-sacrifice, and likewise by her creation of a training system, glorified the nurse's calling, until now, the only striking development that took place was the foundation and establishment of the College of Nursing. It would, indeed, appear that to the throes of war the nurse owes her very existence, and incidentally, directly and indirectly, the Empire owes to the nurse more than ever can be repaid. How many of the millions who throng the Metropolis realise that the noble pile on the Thames Embankment, St. Thomas's Hospital, is in reality, through the Nightingale Nurses' Training School and Home, a national monument to the memory of a single nurse, and to her conviction that a nurse untrained is no nurse? St. Thomas's Hospital fulfilled its mission; but in so doing it accomplished a far greater work. Suffering humanity by hundreds of thousands have found relief within its wards. Yet I venture to assert not one in a million realises that the British Nursing System thus found its genesis.

We have now arrived at another epoch. The world's agony has again brought the nurse to the forefront of public attention, and, chiefly owing to your efforts, the College of Nursing has become a great national and imperial fact, with far-reaching possibilities and potentialities. It is yet in its babyhood; but the baby is stalwart, and is growing in soul and in muscle from day to day. From the outset the College has been assailed from petty and jealous motives, as a big, well-bred dog is assailed by the yelping of mongrel canine way-siders. But the College pioneers have been big enough and great enough to pursue their course despite the din. In contemplating their personal ambitions these objectors have lost sight of the broader issues, and the nurse has become in their esteem a mere pawn in a game of aggrandisement.

It is in this spirit that I offer my criticism. As an interested character I have been watching the proceedings, and to me it seems that certain fundamental matters of policy are not set out in their proper, or, I might say, in their scientific proportion. In a word, I suggest that the economic problem, although by no means forgotten, fails to receive its full measure of consideration, and that the educational problem is apt to occupy undue prominence. The correct order of procedure is reversed. To ensure better conditions of service, better pay, shorter hours of duty, longer holidays, and, above all, to remove the constant anxiety of providing for age and infirmity—these things, it appears to me, are of overwhelming importance. They are the things that make life joyous, and that appeal to every worker more than aught else. In the early days of the history of the College there were many who believed that the economic problem was avoided, or, at all events, indefinitely postponed. The real and the counterfeit champions of the nurse occupied too much time in discussing minor matters. Registration is only comparatively important, and as to the wrangling about the composition of the Registration Authority that is to be, the controversy descended almost to squabbling. After all what does it really matter if there is or is not legislation? Florence Nightingale wrought a miracle without the aid of Parliament, and to-day legislation can accomplish so little that it is not worth taking serious account of. Of one thing there is no room for doubt, no Act of Parliament can effect the economic reforms which I suggest, nor can even help in their acceleration. It has yet to be put on paper and in plain English what material benefit a Registration Act can bring to the working nurse, even if angels sit on the board. The only end that such legislation can achieve is to distinguish the trained from the untrained, and it cannot by any possibility either put the untrained out of employment or limit their sphere of operations, save in respect of public positions from which they are already debarred.

Concerning the better equipment and education of nurses, this I submit will follow as a consequence of improved conditions of service. Make the nurse's life joyous in the full sense, and she will demand more enlightenment and a higher standard of education than even the College proposes to confer. It must be so; it is so in every department of life. If you would have women of talent and of education there must be allurement. The market conditions must be taken account of. Why have we men of genius in the world of letters, of medicine, of surgery, and of law? Simply because there is allurement. Why is there a complaint possible that in the ranks of nursing there is ignorance and illiteracy? Simply because there is a lack of allurement. The most that a hard-working nurse can hope for as the ultimate reward of service for the minority is an old-age State or other pension.

**An Open Letter to Sir Arthur Stanley** (*continued.*)

There are admittedly difficulties in the way—big difficulties. But what great work was ever accomplished where these were absent, and, great as they may be in this instance, they are as nothing in comparison with the mountains that lay in the path of the "Lady of the Lamp." To surmount obstacles is, in my estimation, the true mission of the College of Nursing. Recruits are coming in, but with steady steps and slow. Not ten, but fifty, thousand ought by now to be enrolled. The reason for this is that for thirty years British nurses have been fed on persiflage. The efforts of the prophets have brought them nothing better than a harvest of words. Is it any wonder in these circumstances that there is a tendency to stand aloof? Give them something tangible to seek after, and they will rally to the standard in hosts. They will not only pay a guinea to enter, but, without grudging, a guinea per annum. Lead them, and they will follow. They will do more; they will help to fight the battle. The moment is opportune for earnest and united action. It is the nurse's hour: it is woman's hour.

These suggestions I take the liberty of submitting with great respect for your consideration, and in so doing I venture to suggest that the impartial views of those concerned might afford substantial help in forming a public opinion on the subject in the nursing world. It is eminently desirable that the working nurse should articulate. In this connection I may use Shakespeare's words:—

> Send us to Rome
> The best, with whom we may articulate
> For their own good and ours.

IERNE.

